# A Randomized Controlled Trial of a Theory-Informed School-Based Intervention to Prevent Waterpipe Tobacco Smoking: Changes in Knowledge, Attitude, and Behaviors in 6th and 7th Graders in Lebanon

**DOI:** 10.3390/ijerph15091839

**Published:** 2018-08-26

**Authors:** Rima Nakkash, Tamara Lotfi, Dima Bteddini, Pascale Haddad, Hala Najm, Lina Jbara, Hala Alaouie, Lama Al Aridi, Ahmad Al Mulla, Ziyad Mahfoud, Rima A. Afifi

**Affiliations:** 1 Department of Health Promotion and Community Health, Faculty of Health Sciences, American University of Beirut, Beirut 1107-2020, Lebanon; rn06@aub.edu.lb (R.N.); db15@aub.edu.lb (D.B.); ha118@aub.edu.lb (H.A.); 2 Department of Epidemiology and Population Health, Faculty of Health Sciences, American University of Beirut, Beirut 1107-2020, Lebanon; tamara_loutfi@hotmail.com; 3 Independent consultant, Dusseldorf 40210, Germany; pascalehaddad84@gmail.com; 4 Soins Infirmiers et Développement Communautaire (SIDC), Sin El Fil 1100, Lebanon; hnajm@sidc-lebanon.org; 5 Academic Coordinator, Sorbonne University Abu Dhabi, 38044 Abu Dhabi, United Arab Emirates; lina.a.jbara@gmail.com; 6 Independent consultant, Aley 1501, Lebanon; lamaalaridi@gmail.com; 7 Department of Medicine, Hamad Medical Corporation, Doha 4147, Qatar; ALMULLA@hamad.qa; 8 Department of Global and Public Health, Weill-Cornell Medicine, Doha 24144, Qatar; zrm2001@qatar-med.cornell.edu; 9 Department of Community and Behavioral Health, College of Public Health, University of Iowa, Iowa City, IA 52242, USA

**Keywords:** waterpipe, intervention, Lebanon, school, evaluation, RCT

## Abstract

Waterpipe tobacco smoking (WTS) is spreading worldwide. Research has indicated health consequences of WTS similar to cigarettes. Prevalence of WTS is high among young people. In Lebanon, current use rates of 35% have been documented among 13–15 year olds. We evaluated a school-based intervention. *Method*: We conducted a randomized-controlled-trial of a theory-informed WTS intervention. The intervention consisted of ten sessions based on social cognitive theory and the social influences approach. Thirty-one schools participated: 14 intervention and 17 control; a total of 1279 students completed pre and post assessments. We measured knowledge, attitudes and self-reported behaviors related to WTS using Chi-square tests and regression analyses to compare results between the two study arms. *Results*: The intervention increased knowledge of intervention group compared to control group participants—about WTS constituents and health consequences; and shifted attitudes of intervention group participants to be even more unfavorable towards WTS. We found no impact of the intervention on WTS behaviors. *Discussion*: The effectiveness of the intervention on knowledge and attitudes supports previous research. The lack of intervention effect on behavior is not surprising given the timing of the post assessment immediately after the intervention, and the social context that was supportive of waterpipe use.

## 1. Introduction

Despite misconceptions that it is safer than cigarettes [1,2], waterpipe tobacco smoking (WTS) has been linked to a variety of short term and long-term health consequences, including respiratory illness, cancer, cardiovascular diseases, and mental ill health [3,4,5,6,7,8]. Because of the evidence of health effects, the World Health Organization first released a WTS advisory note in 2005, and recently released an updated version [9]. WTS has become pandemic, with use documented in every continent [10,11,12,13,14]. Prevalence is especially high among young people [13,14,15]. In some global regions such as the Eastern Mediterranean region, current WTS rates (smoking at least once in the past month) reach 37% among 13–15 year olds [16,17]. Generally, WTS patterns among young people indicate mostly intermittent use; with 55%–78% reporting WTS once a month or less [14,18,19,20].

In Lebanon, the high prevalence among increasingly younger age groups is alarming and calls for immediate intervention. The Global Youth Tobacco Survey (GYTS) conducted in 2011 with a nationally representative sample of youth (13–15 years old) in grades 7–9, indicated that 34.8% of students were current waterpipe smokers with no differences by gender [21]. In addition, comparative data between the GYTS in Lebanon in 2001 and 2005 indicates a decrease in prevalence of cigarette smoking but an increase in prevalence of WTS [22]. In terms of patterns of use, recent evidence from a longitudinal study of school children (mean age 14 years) indicates that among current waterpipe tobacco smokers, 47% smoked less than once per week, 38% weekly but not every day, 16% everyday/almost every day [23].

A variety of factors have been found to influence WTS among youth [1,13,24]. Akl et al. [1] found that positive attitudes and misperceptions about harm were associated with increased WTS. In addition, peer pressure as well as family influences increased WTS. The absence of organizational—including school-based—and national policy also increased WTS. Best practices for tobacco control suggest the importance of intervention at individual interpersonal, organizational, community and policy levels [25,26,27,28]. The best practices described above are a result of decades of research on cigarette smoking. Though WTS is a form of tobacco use, several aspects differentiate it from cigarette smoking including toxicants, patterns of smoking, social norms, policy environments, and the extent of current evidence [24]. These differences likely necessitates adaptation rather than adoption of cigarette-based tobacco-control interventions to waterpipe. A recent review of WTS interventions evaluated to date stated that “there is a lack of evidence of effectiveness for most waterpipe interventions”[29].

School-based smoking prevention programs have been a central component of comprehensive tobacco control strategies [30,31,32,33]. There is mixed evidence on the efficacy of school-based cigarette smoking prevention interventions. An analysis of 49 randomized controlled trials (RCT) (including over 140,000 school children) aiming to prevent children who had never smoked cigarettes from becoming smokers found an average 12% reduction in smoking initiation for the intervention compared to the control groups at greater than one year post intervention [34]. However, no overall effect was detected at one year or less. The effectiveness of interventions in schools has been found to be further enhanced by school policies that enforce nonsmoking on campus [35,36]. When assessing intervention approaches, the combined social competence and social influences interventions were found to be more effective than other programs [34]. Social competence interventions focus on enhancing life skills (such as decision making), cognitive skills (such as refusal skills), and self-esteem/control; while social influence interventions focus on enhancing awareness of the social influences on tobacco use, and providing skills to resist peer pressure. Other studies have indicated that school-based programs to prevent and control tobacco use have limited effects in the absence of other community and policy interventions [32,37,38], highlighting the need for a comprehensive approach to tobacco control [39].

Individual or group interventions to prevent or control youth WTS are still scant [29], but have shown generally positive results. Anjum et al. [40] assessed the impact of a school-based education intervention on knowledge, attitudes and behaviors of 14–18 year olds students (mean age; 15 years) using a pre post-test design. They found significant changes in knowledge and attitudes at post-test (two months after initiation of intervention) as compared to pretest; no significant differences were found in behavior. Stamm-Balderjahn et al. [41] tested the impact of an education intervention targeting high school and vocational school students using a quasi-experimental pre post-test design. At 6-months post-intervention, non-smoking students in the intervention groups were significantly less likely to have taken up waterpipe smoking than those in the comparison group; no differences by group were seen in those who had already been smoking at baseline. Quadri et al. [42] tested the impact of an educational intervention provided to high school and college students in class (mean age; 20 years) on knowledge of oral health consequences of tobacco use; and found increases in knowledge pre and post intervention. In a pre-posttest design, Essa-Haddad et al. [43] tested the impact of a tailored web-based intervention on tobacco use directed at college students (mean age; 25 years) and found no increases in knowledge but statistically significant decreases in waterpipe smoking one-month post intervention.

Perhaps the most methodologically and theoretically robust study to date was conducted by Lipkus et al. [44]. Researchers conducted a randomized pre-posttest control group design to test the impact of an online intervention directed at college students (mean age; 20 years) and focused on receiving information about waterpipe harm and exposure. They found significant increases in the intervention group in perceived and factual knowledge, in perceptions of risk and worry about harm and addiction, and in reported desire to quit immediately after receiving this information intervention compared to comparison group. Further, the effect of receiving information on desire to quit was mediated by changes in attitudes (perceptions of harm and worry). In a similar follow-up study, Mays et al. [45] tested the effectiveness of an online intervention by randomizing young adults (mean age; 25 years) to three conditions: control, information about waterpipe harm, and information about waterpipe harm and addiction. Immediately post intervention, those who had been randomized to the harm condition had significant increases in perception of the harm and addictiveness of WTS and in desire to quit as compared to the control condition. There were no differences between the harm and addiction conditions. Islam et al. [46] tested the impact of text and pictorial health warnings placed on the waterpipe and its accessories on motivation to stop or reduce smoking of ever and current WTS college students (mean age; 22 years). Ever users were significantly more likely to be motivated to stop or reduce smoking than current users for almost all pictorial warnings and some text warnings. Sutfin et al. [47] tested the impact of point-of-sale communication about the constituents in waterpipe tobacco smoke. Their messages were developed based on theoretical constructs and pilot tested prior to use. All the messages resulted in an increase in perceptions of health risks and addiction. The main limitations of the above studies include lack of representative samples (convenient or purposive samples); methodologically weaker designs (quasi experimental design, no randomization, only post-test measures); and few theoretically-driven intervention designs. Interventions varied in exposure time but were mostly brief (one time online exposure, presentations, 2hr face-to-face lectures, brochure etc,) and mostly composed of knowledge rather than skill building. Intervention sites varied: school, hospital, point of sale, web or online, lab; and not all were waterpipe specific interventions.

With the above limitations in mind, we developed a WTS intervention that was theory-based, with higher intervention dose than previous interventions; and tested its effectiveness in changing knowledge, attitude and behavior, using a robust RCT evaluation design of a nationwide representative sample of school students in 6th and 7th grades in Lebanon. This paper describes the intervention, its evaluation design, and results. Since enrollment in school in Lebanon is high, reaching 98% and 81% respectively for age groups 6–11 years and 12–17 years [48], schools are a critical site for intervention. A ministerial decree banned smoking in Lebanese schools as far back as 1993, and a comprehensive tobacco-control law—which included bans on smoking in all public places such as schools—was enacted in 2011. However, enforcement of these policies remains variable. Education about the consequences of tobacco use is also required as part of the National curriculum for all schools in Lebanon, but is not implemented comprehensively. In this very lax policy environment, with the realistic assessment that effective enforcement was not likely in the near term, we chose to develop and implement a school-based intervention despite the evidence of general lack of effectiveness of school-only interventions for cigarette smoking prevention. Our decision was based on the epidemic of WTS among an increasingly young age group, a commitment to the right to information, and to enhancing knowledge and skills using the most up-to-date knowledge and approach. We hypothesized that the intervention would lead to changes in knowledge, attitudes and behavior of the students randomized to the intervention as compared to their peers in the control group.

## 2. Methods

This study is a randomized controlled trial [49] of a school-based intervention to prevent waterpipe use, which was conducted in Lebanon during the 2011–2012 academic year.

### 2.1. Sample Size

This Lebanon RCT was a part of a larger research project that also included a similar intervention in Qatar [50]. The sample size (for both countries) was calculated based on the following assumptions: the estimated mean baseline value of waterpipe use (50% in Lebanon [22]); a decrease of 10% in the absolute prevalence of waterpipe use among students randomized to the intervention group; a *p*-value of 0.05; a power of 80%, a loss to follow-up (drop-out) of 15%; and an intra-class correlation of 0.03 based on an earlier intervention conducted in Lebanon among school children in 5th and 6th grades attending United Nations Refugee and Works Agency for Palestine (UNRWA) schools in Burj El Barajneh camp of Beirut. Due to the significantly higher prevalence of current waterpipe use among this age group in Lebanon, the sample size needed in Lebanon was less than that needed in Qatar. However, for comparability purposes, equal sample sizes were taken in both countries, with the requirement being the minimum needed in Qatar (n = 2600).

The sampling frame included all public and private Lebanese schools registered at the Ministry of Education and Higher Education (MEHE) across all Lebanese Governorates and enrolling at least 60 students in grade 6 and/or grade 7. Based on the above assumptions, and on the total number of students and proportion of public versus private education in Lebanon, the target sample size was 40 schools, with a distribution of 24 private versus 16 public schools.

### 2.2. Randomization

Forty schools were selected by stratified random sampling according to the proportion of public vs private school students enrolled in each region (strata). Selected schools were then randomly assigned into intervention or control groups, prior to inviting them to participate. Schools in intervention group received ten sessions to prevent waterpipe use, while schools in control group didn’t receive any sessions. Students in control schools received a pamphlet after pre-assessment data collection on harmful effects of waterpipe smoke on the human body.

Schools were invited to participate in August–September 2011. When a selected school refused to participate, we randomly selected another one from the same region, and assigned it to the same group as the one originally selected. Overall, we invited 50 schools to participate, out of which 32 agreed to participate (16 private, and 16 public). Of these 32, 15 had been randomly assigned to the intervention group and 17 to the control group—i.e., there were more refusals in the schools assigned to intervention groups despite the fact that schools were blinded to group before they agreed. One intervention school dropped out after receiving the first intervention session. These 31 schools included 17 (55%) in urban areas, 16 (52%) private, and 25 (81%) mixed gender. Within participating schools, class sections were randomly selected to ensure about 60 students per school. Eligibility criteria for participants in the intervention trial were therefore that they were 6th or 7th graders in one of the eligible and participating schools randomly selected from the MEHE roster. A total of 2142 6th and 7th grade students (mean age: 12.27; SD: 1.12) were sampled from these schools: parental consent was granted for 1658 students (response rate = 77.4%), out of which 1622 students agreed to participate (response rate = 97.7%). One intervention school and one control school were lost to post-test follow-up leaving 13 intervention schools and 16 control schools from which data was collected at post-test.

### 2.3. Intervention

The intervention consisted of ten sessions carried out over 5 months (January–May 2012) on school premises and during school hours. The intervention was implemented by 6 trained facilitators using an intervention manual to ensure consistency. In line with best practices, intervention sessions focused on a social influence approach; used social cognitive theory constructs [51]; and were interactive rather than didactic. The ten sessions of the intervention were organized as follows: four sessions were knowledge-based, and the other six were skill-based, including: media critical analysis, decision-making skills, refusal skills and social promise. More information on the intervention can be found elsewhere [52].

### 2.4. Outcomes

**Figure 1:**
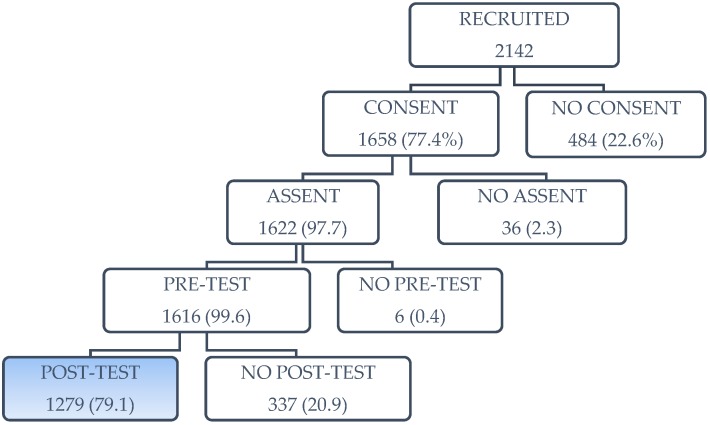
Flow chart of participants.

Behavioral, attitudinal, and knowledge-related outcomes were measured through self-administered surveys completed by participating students during school hours at 2 points in times: time 1 (pre-intervention) and time 2 (immediately post intervention). A pre-test (baseline measurement) was administered to all participating students prior to randomization. Immediately following the intervention, a post-assessment was administered to all participating students of schools in both intervention and control groups. The post-assessment was identical to the baseline, except for one section administered to the intervention group students that included process evaluation questions. A unique identifier was assigned to each participating student for the purpose of linking the pre-tests to the post-assessments; however, no names appeared on the survey. Figure 1 shows the participant flow from recruitment to post-assessment.

### 2.5. Variables

The survey included 4 sections: socio-demographics, knowledge, attitudes, and behaviors. The development of the survey has been described previously [53,54]. We describe only those variables from each section that are used in the current analysis. The section on socio-demographics included age, sex, governorate, student’s self-reported school performance (below average of students in my class, average, above average), self-reported family socio-economic status (much poorer & poorer, no difference, wealthier & much wealthier than others your age), grade level (6th or 7th grade), and school type (private and public). Participants were asked 15 true/false knowledge questions related to the effects of waterpipe or constituents of waterpipe tobacco smoke; and 13 agree/disagree attitude questions. For behaviors, the survey included questions about whether each parent smoked cigarettes and/or waterpipe. The survey also asked about whether the participants had ever smoked waterpipe; were current waterpipe smokers (smoked at least once in the last month); and had attempted to quit smoking waterpipe (only asked of those who responded that they had smoked waterpipe at least once in the last month). The survey was available in Arabic and English and each school chose the language they preferred.

### 2.6. Blinding

At the time of recruitment, all school administrations, parents, and students were kept blinded to the group assignments, i.e. whether the school belonged to the intervention or to the control group. Data collectors (who were the intervention implementation team) for the pre-assessment were also blinded. At post-assessment, data collectors were not blinded—however, the implementation team switched schools, so none conducted the post-assessment at a school where they had implemented the intervention.

### 2.7. Ethical Considerations

This study was granted approval from the MEHE and Institutional Review Board at the American University of Beirut, Lebanon (FHS-RA-11). Approvals were also obtained from schools’ principals, and consent and assent from the parents and students respectively.

### 2.8. Analysis

Demographic and baseline characteristics of students and their schools were summarized and compared between the two study arms using the Chi-squared test except for students’ age where the independent t-test was used.

Between study arms differences in students’ answers on knowledge, attitude and behavioral questions at baseline and then post intervention were compared using the chi-squared or Fisher’s exact test when needed and were also adjusted for the possible clustering effect for students within each school.

The McNemar test was used to assess changes on the outcome measures at post intervention as compared to baseline values within each study arm (results not shown here but available from authors).

Increase in knowledge (more knowledgeable about the harms of WTS) and attitude (more negative attitude towards WTS) scores were computed by taking the post minus pre sum of total correct answers on the knowledge questions or total desirable answers on the attitude questions. For each question answered correctly a point was given, and zero otherwise (whether it was a wrong answer or “I don’t know”). Those scores were compared between the intervention and control arm using simple linear regression, and differences±standard errors are presented with two the p-values adjusted for clustering. Those differences were also adjusted for the demographics variables using multiple linear regression and results are presented in a similar manner as described above.

The logistic regression model was used to assess the difference in each of the behavior questions between the two study arms in a univariate fashion and then adjusting for possible clustering and baseline demographic values using multivariate logistic regression. Unadjusted and adjusted OR along with their 95% confidence intervals are presented in addition to p-values adjusted for clustering.

Participants who answered less than 40% of the questions were excluded (n = 343). All analyses were done using STATA (version 12, College Station, TX, USA) and IBM-SPSS (version 22, Armonk, NY, USA) and a *p*-value of 0.05 or less was considered statistically significant.

## 3. Results

Overall, 1279 students (hereafter called participants) completed the pre and post assessment. Participants were almost equally distributed among the intervention (52.6%) and control (47.4%) groups. About half (51.3%) were female, in public schools (49.4%), and in grade 7 (53.2%). Participants came from all the Lebanese governorates. The majority of our participants perceived that they had average grades (65.9%), and that their family socioeconomic status was the same as others their age (71.4%) (Table 1). There were no differences between intervention and control group participants on most socio-economic characteristics. Only governorate and grade levels were significantly different between the two groups (Table 1).

### 3.1. Effects of the Intervention on Knowledge Regarding WTS

At pre-assessment, there were no differences in knowledge between the participants from the intervention and control groups, except in one question assessing knowledge of bladder cancer as a consequence of WTS. This suggests group equivalence in knowledge at pre-assessment. Generally, over 75% of the participants in both groups correctly identified lung cancer, respiratory and heart problems as consequences of WTS. And about 75% of the participants in both groups correctly denied that the fruit in the waterpipe tobacco make it a healthier choice; and that the water in the waterpipe bowl helps clean the toxic substances. Only about 50% of the participants in both groups correctly stated that WTS was addictive, and that waterpipe tobacco has nicotine in it. Other results are shown in Table 2.

Conversely, at post-assessment, knowledge of intervention participants was significantly higher than that of control participants on 11 of the 15 knowledge questions after adjusting for the possible clustering effect in each school, and not different on three. Over 90% of intervention group participants correctly stated that WTS can cause lung cancer, oral cancer, heart and respiratory problems and addiction; and that waterpipe tobacco has nicotine in it. Other results are shown in Table 2.

### 3.2. Effects of the Intervention on Attitudes Regarding WTS

At pre-assessment, there were no differences in attitudes between the participants in the intervention and control group, except for one item. This suggests group equivalence in attitudes at pre-assessment. Attitudes were generally not favorable to WTS. Close to or over 70% of participants in both groups agreed that smoking the waterpipe is a bad habit, and disagreed that if they smoked waterpipe, they would have more friends or would be more attractive. However, only about half of both groups agreed if they smoke waterpipe, they could get addicted to it; or would be more likely to get oral cancer when they grow up than if they do not smoke waterpipe. The only significant difference in attitudes between the two groups was in relation to difficulty in quitting. More of the intervention group participants than control group participants agreed that “once someone has started using the waterpipe, it is difficult to quit.” More results shown are shown in Table 3.

At post-assessment, three (out of 13) of the attitude items were significantly different between the two groups after adjusting for the possible clustering effect within each school, with the intervention group having more negative attitudes towards WTS on these items than the control group. Intervention participants were significantly more likely to agree that if they smoke waterpipe, they could get addicted, and that they are more likely when they grow up to get lung, oral, and bladder cancer as well as heart problems than if they do not smoke it. More results are shown in Table 3.

### 3.3. Effects of the Intervention on WTS Behavior

The survey asked 3 questions about WTS behavior: ever use, current use (use at least once in the last month), and quit attempts. None of these questions was significantly different at pre-assessment between intervention and control group participants. Approximately 43% of 6th and 7th grade participants in both groups had ever tried a waterpipe; about 37% had smoked a waterpipe at least once in the last month; and about 42% had tried to quit smoking waterpipe (data not shown).

Similarly, at post-assessment, there was no significant difference in behavior between the intervention and control group participants on these items (data not shown).

Table 4 summarizes the impact of the intervention by comparing knowledge, attitudes, and behaviors of the intervention group as compared to the control group. The summary variable for knowledge and attitude indicates significant difference in post test knowledge and attitudes between the intervention and control group participants controlling for pretest knowledge and attitudes respectively. The table also confirms the null impact of the intervention on behaviors after adjusting for the clustering effect.

## 4. Discussion

This study described the effectiveness of a RCT of a nationally-implemented school-based waterpipe tobacco smoking prevention intervention in Lebanon. Our sample of 6th and 7th graders are the youngest to date to be participants in research about the effectiveness of an intervention to prevent and control WTS. Our results suggest that the intervention was successful in enhancing knowledge of intervention group participants—as compared to control group participants—about WTS constituents and health consequences; and in shifting attitudes of intervention group participants to be even more unfavorable towards WTS. We found no impact of the intervention on WTS behaviors.

The effectiveness of the intervention on knowledge and attitudes supports previous scholarship on the impact of individual and group intervention to prevent or control waterpipe use. Positive effects were found in almost all interventions which measured changes in knowledge [40,42,44]. Essa-Haddad et al. [43] attributed their lack of impact on knowledge change to universally high knowledge of the consequences of tobacco use at baseline. Although the specific items they used on the survey were not included in their publication, the text of their publication suggests that the knowledge items on the survey may have been general to various tobacco types, and not specific to waterpipe, which may also explain their results. Attitudes and perceptions of risk were also positively impacted by previous interventions [40,44,45,46,47]. The surprising finding in our intervention was the already high negative attitude towards waterpipe smoking exhibited by our participants pre-intervention. The intervention was able, however, to enhance those attitudes even further.

The lack of intervention effect on behavior change is not surprising. In fact, students in both the intervention and control group were more likely to report waterpipe smoking in the past month at post assessment as compared to pre-assessment. This is somewhat expected. Evidence from epidemiological trend data [55] as well as experimental intervention reports [56,57] indicate that smoking prevalence increases throughout the school year. Previous waterpipe interventions that have found effect on behavior change were implemented with older students and measured behavior at least 2 months following intervention [41,43]. The study that most closely matched our participants in terms of age group [40] showed no impact on behavior 2 months after the initiation of the intervention. More broadly, a recent substance use intervention based on the social-influences model and implemented in 7 European countries and 70 schools with students of similar ages to those in our intervention (12–14 year olds) found no intervention effect on cigarette use (quitters or reducers) among cigarette smokers, although the intervention was able to decrease alcohol and cannabis risk behaviors when measured 9 months after pre-assessment [57].

We assessed behavior immediately following the intervention. Behavioral theory suggests that behavior is preceded by changes in knowledge and attitudes [58], and that this temporality may require days, weeks, or months to detect. Although our original protocol included a 6-month follow-up, various delays in implementation resulted in an inability to collect data at follow-up. More importantly, ecological models generally [59], and best practices in tobacco control more specifically [25,26,27], underscore the importance of comprehensive multi-level programs—including supportive environments—to promote behavior change. The context in which the current intervention was implemented is antithetical to a supportive environment. A variety of contextual factors affected implementation, including factors at the country level, at the school level, and related to social norms around waterpipe smoking. At the country level, during the time of the intervention, political unrest made it difficult to implement the intervention both for practical reasons (reaching schools) as well as relevance reasons—WTS as a topic sometimes seemed trivial to discuss amidst daily living struggles and concerns of students. We faced school-based contextual challenges such as traditional teaching methods, structural barriers (lack of electricity), and family issues that students were dealing with. Finally, the socio-normative environment of tobacco use in Lebanon is quite supportive of tobacco use, and particularly waterpipe. Students told us of school trips where they were offered and smoked waterpipes. Despite a policy prohibiting smoking in schools, many schools allow smoking on their premises. We have described these contextual challenges to this intervention more fully previously [52]. All the above contextual factors make efforts to effectively message around tobacco prevention and control difficult; and suggest that we may not have found changes in behavior even with a follow-up assessment. In context of high uncertainty and/or of extreme disadvantage, and which lack policy/regulatory attention to tobacco control, it is unclear what combination of interventions will be most impactful in controlling youth tobacco use. More research, dialogue and analysis are needed in the public health community to strengthen the evidence base in these types of settings.

The strengths of this intervention include the robust RCT evaluation design; a nationally representative sample; and that the intervention was guided by health behavior theory. Limitations include the lack of a follow-up assessment that might have been able to capture changes in behavior. In addition, our sample was slightly skewed towards participants from public schools. In Lebanon, 60% of students attend private schools. Our sample is more evenly distributed between public and private school students, given the higher refusal rate of private schools that were selected to participate. Our measures of behavior were self-reported but self-report has been shown to be a valid indicator of youth tobacco use [60]. Also, for some of the knowledge questions, the ‘correct’ answer was ‘no’ (e.g. ‘The fruits in the tobacco make the waterpipe a healthier choice’). However, those came towards the end of the knowledge questions, and it is possible that students answered ‘yes’ without carefully reading. Finally, social desirability is always a possible bias in research. We attempted to control the extent of this bias by ensuring that the data collectors were different from the implementation team at post-test—as described in the blinding section above.

Future research should evaluate other intervention approaches such as community mobilization, counter-messaging and waterpipe specific mass media campaigns and document their effectiveness in changing social norms around WTS and denormalizing WTS. For practice, and policy, enforcement of the WHO MPOWER strategies should work synergistically to create environments supportive of behavior change related to WTS [61]. WHO MPOWER strategies include monitoring tobacco use and prevention policies; protecting people from tobacco smoke by enforcing public smoking bans; offering cessation services; warning about the dangers of tobacco mainly through health warnings on tobacco packages; enforcing bans on tobacco advertising, promotion and sponsorship; and fiscal policies to curb tobacco use. Regular surveillance in countries where MPOWER measures are enforced provide opportunities to test effectiveness of the various strategies in curbing WTS among youth and other vulnerable populations.

## 5. Conclusion

Evidence-informed school-based waterpipe prevention interventions that integrate behavior change theory and social-influences approaches are effective in changing knowledge and attitudes of young persons. Their ability to impact behavior is less clear, particularly in difficult contexts, and where social norms tend to be pro smoking. More research is needed to understand what combination of interventions work best in these contexts for comprehensive tobacco control.

## Figures and Tables

**Table 1:** Descriptive Socio-demographic Characteristics of the participants overall, and in intervention and control groups.

Variable		Total	Intervention Gp.	Control Gp.
		% (N)	% (N)	% (N)
Group (N = 1279)	Control	47.4% (606)		
	Intervention	52.6% (673)		
Age (N = 1267)	Mean (SD)	12.27 (1.12)	12.26 (1.11)	12.28 (1.13)
Sex (N = 1264)	Male	48.7% (615)	48.4% (322)	48.9% (293)
	Female	51.3% (649)	51.6% (343)	51.1% (306)
Governorate (N = 1279) *	Beirut	12.4% (159)	15.2% (102)	9.4% (57)
	Bekaa	14.9% (190)	19.9% (134)	9.2% (56)
	Mount Lebanon	25.9% (331)	21.5% (145)	30.7% (186)
	Nabatieh	8.0% (103)	8.3% (56)	7.8% (47)
	North	28.2% (361)	27.2% (183)	29.4% (178)
	South	10.6% (135)	7.9% (53)	13.5% (82)
Student’s self-reported school performance (N = 1219)	Below average	3.4% (42)	3.4% (22)	3.5% (20)
	Average	65.9% (803)	65.9% (425)	65.8% (378)
	Above average	30.7% (374)	30.7% (198)	30.7% (176)
Socioeconomic Status (N = 1173)	Much poorer & poorer	5.5% (64)	4.7% (29)	6.3% (35)
	No difference	71.4% (838)	71.5% (444)	71.4% (394)
	Wealthier & much wealthier	23.1% (271)	23.8% (148)	22.3% (123)
Grade Level (N = 1279)*	6th grade	46.7% (598)	43.7% (294)	50.2% (304)
	7th grade	53.2% (681)	56.3% (379)	49.8% (302)
School type (N = 1279)	Public	49.4% (632)	49.9% (336)	48.8% (296)
	Private	50.6% (647)	50.1% (337)	51.2% (310)
Parents smoke cigarettes (N = 1234)	At least one parent	62.6% (768)	64.9% (417)	60.1% (351)
	Neither parent	37.4% (458)	35.1% (225)	39.9% (233)
Parents smoke waterpipe (N = 1256)	At least one parent	43.6% (544)	43.1% (290)	44.1% (254)
	Neither parent	56.4% (704)	55.9% (368)	56.9% (336)

*p < 0.05 for difference between intervention and control groups.

**Table 2:** Differences in WTS knowledge between intervention and control group participants at pre-assessment and post-assessment.

	Pre-Assessment (% Yes)			Post-Assessment (% Yes)		
Variable	Intervention	Control	*p*-Value ^†^	Intervention	Control	*p*-Value ^†^
Waterpipe smoking can cause lung cancer.	74.80%	78.85%	0.286	93.40%	80.65%	<0.001 *
Waterpipe smoking can cause oral (mouth) cancer.	44.39%	43.41%	0.840	90.09%	65.63%	<0.001 *
Waterpipe smoking can cause bladder cancer.	32.28%	26.89%	0.213	71.91%	43.18%	<0.001 *
Waterpipe smoking can cause heart problems.	77.61%	81.47%	0.080	91.51%	81.31%	<0.001 *
Waterpipe smoking can cause respiratory problems.	80.16%	81.63%	0.646	90.48%	83.36%	0.009 *
Waterpipe smoking can cause addiction.	61.09%	57.58%	0.481	90.59%	69.91%	<0.001 *
Waterpipe tobacco has nicotine in it.	52.12%	51.26%	0.879	90.73%	60.24%	<0.001 *
Waterpipe tobacco has dangerous chemical substances in it.	62.32%	63.83%	0.664	85.14%	66.96%	<0.001 *
The waterpipe smoke has carbon monoxide in it.	39.58%	42.60%	0.563	72.07%	43.81%	<0.001 *
The fruits in the tobacco make the waterpipe a healthier choice.	15.02%	14.95%	0.986	19.56%	12.46%	0.011 *
The water in the bowl helps clean the waterpipe from toxic substances.	28.01%	24.14%	0.358	30.02%	23.86%	0.092
Using a filter will prevent the exposure to toxic substances.	35.23%	34.90%	0.929	37.75%	33.51%	0.234
Using a mouth piece will prevent transfer of bacteria.	34.33%	35.56%	0.760	42.35%	39.89%	0.406
Sharing the waterpipe can cause the transfer of infectious diseases from one person to the other.	62.94%	65.36%	0.458	76.53%	68.33%	0.019 *
Smoking waterpipe around people who are not smoking puts them at risk of health problems.	66.29%	64.73%	0.569	76.13%	69.07%	0.031 *

^†^ adjusting for the possible clustering effect within each school; * significant differences between intervention and control.

**Table 3:** Differences in WTS attitudes between intervention and control group participants at pre-assessment and post-assessment.

	Pre-assessment (% Agree)			Post-assessment (% Agree)		
Variable	Intervention	Control	*p*-Value ^†^	Intervention	Control	*p*-Value ^†^
Smoking waterpipe is a bad habit.	83.76%	83.19%	0.865	86.50%	84.21%	0.458
Once someone has started using the waterpipe, it is difficult to quit.	57.45%	63.62%	0.103	69.20%	65.69%	0.359
If I smoke waterpipe, I would relax.	29.37%	32.10%	0.277	26.57%	30.12%	0.376
If I smoke waterpipe, I would have more friends.	21.99%	25.09%	0.340	23.03%	23.85%	0.847
If I smoke waterpipe, I would be more attractive.	30.35%	30.00%	0.927	27.30%	28.06%	0.842
If I smoke waterpipe, I would have a good time.	33.22%	33.99%	0.829	28.85%	35.63%	0.139
If I smoke waterpipe, I can get addicted to it.	52.76%	51.50%	0.792	69.60%	56.26%	0.009 *
If I smoke waterpipe, there is a chance that my hair, clothes or breath will smell.	65.62%	60.88%	0.265	75.59%	71.45%	0.297
I’m more likely to have lung cancer when I grow up if I smoke waterpipe than if I don’t.	59.46%	63.44%	0.331	72.58%	65.30%	0.055
I’m more likely to have oral (mouth) cancer when I grow up if I smoke waterpipe than if I don’t.	43.52%	46.42%	0.393	68.35%	55.75%	0.001 *
I’m more likely to have bladder cancer when I grow up if I smoke waterpipe than if I don’t.	33.01%	34.88%	0.602	57.42%	42.76%	<0.001 *
I’m more likely to have heart problems when I grow up if I smoke waterpipe than if I don’t.	58.08%	60.89%	0.404	68.83%	60.31%	0.057
If I smoke there’s a chance that my teeth color might change with time.	65.97%	68.96%	0.172	80.94%	73.10%	0.075

^†^ adjusting for the possible clustering effect within each school; * significant differences between intervention and control.

**Table 4:** Improvement in knowledge and attitudes scores.

Variable	Intervention-Control (Unadjusted) Mean±SE	*p*-Value ^†^	Intervention-Control (Adjusted) ^‡^ Mean±SE	*p*-Value ^†^
More knowledgeable about the harms of WTS	2.12 (0.30)	<0.001 *	2.17 (0.25)	<0.001 *
More negative attitude towards WTS **	0.75 (0.24)	0.031 *	0.87 (0.21)	0.005 *
	**OR (95% CI)**	***p*-Value ^†^**	**Adjusted OR (95% CI)**	***p*-Value ^†^**
Have you ever tried waterpipe (even one or two inhalations)?	0.881 (0.701, 1.106)	0.631	1.070 (0.815, 1.404)	0.751
Did you smoke waterpipe at least once in the last month?	1.043 (0.743, 1.466)	0.836	0.906 (0.591, 1.389)	0.484
Have you tried to stop smoking waterpipe?	0.733 (0.476, 1.13)	0.331	0.554(0.321, 0.954)	0.052

^†^ adjusting for the possible clustering effect within each school; ^‡^ adjusted for all demographic variables in Table 1 in addition to the baseline value of that domain. For example, for increase in knowledge, the model was also adjusted for baseline knowledge. * significant differences between intervention and control. ** the odds of having negative attitudes towards WTS among intervention is 0.75 times that among control group. This means that the control group had more negative attitudes than the intervention group towards WTS.

## References

[1] Akl E.A., Ward K.D., Bteddini D., Khaliel R., Alexander A.C., Lotfi T., Alaouie H., Afifi R.A. (2015). The allure of the waterpipe: A narrative review of factors affecting the epidemic rise in waterpipe smoking among young persons globally. Tob. Control.

[2] Salloum R.G., Abu-Rmeileh N., Hamadeh R., Thomas J., Mostafa A., Yusufali A., Kheirallah K.A., Macauda M.M., Theis R.P., El Kadi L. (2017). Policy-Relevant Context of Waterpipe Tobacco Smoking among University Students in Six Countries Across the Eastern Mediterranean Region: A Qualitative Study. Asian Pac. J. Cancer Prev..

[3] Alomari M.A., Al-Sheyab N.A. (2018). Impact of waterpipe smoking on blood pressure and heart rate among adolescents: The Irbid-TRY. J. Subst. Use.

[4] El-Zaatari Z.M., Chami H.A., Zaatari G.S. (2015). Health effects associated with waterpipe smoking. Tob. Control.

[5] Mamtani R., Cheema S., Sheikh J., Al Mulla A., Lowenfels A., Maisonneuve P. (2017). Cancer risk in waterpipe smokers: A meta-analysis. Int. J. Public Health.

[6] Montazeri Z., Nyiraneza C., El-Katerji H., Little J. (2017). Waterpipe smoking and cancer: Systematic review and meta-analysis. Tob. Control.

[7] Ramji R., Arnetz B.B., Nilsson M., Wiklund Y., Jamil H., Maziak W., Arnetz J. (2017). Waterpipe use in adolescents in Northern Sweden: Association with mental well-being and risk and health behaviours. Scand. J. Public Health.

[8] Waziry R., Jawad M., Ballout R.A., Al Akel M., Akl E.A. (2017). The effects of waterpipe tobacco smoking on health outcomes: An updated systematic review and meta-analysis. Int. J. Epidemiol..

[9] World Health Organization (2005). TobReg—Advisory Note: Waterpipe Tobacco Smoking: Health Effects, Research Needs and Recommended Actions by Regulators.

[10] Abdullah P., Costanian C., Khanlou N., Tamim H. (2017). Prevalence and characteristics of water-pipe smoking in Canada: Results from the Canadian Tobacco Use Monitoring Survey. Public Health.

[11] Combrink A., Irwin N., Laudin G., Naidoo K., Plagerson S., Mathee A. (2010). High prevalence of hookah smoking among secondary school students in a disadvantaged community in Johannesburg. S. Afr. Med. J. Suid-Afrik. Tydskr. Vir Geneeskd..

[12] Greenhalgh E.M., Bayly M., Winstanley M.H., Scollo M.M., Winstanley M.H. (2017). Prevalence of use of different types of tobacco product. Tobacco in Australia: Facts and Issues.

[13] Maziak W., Taleb Z.B., Bahelah R., Islam F., Jaber R., Auf R., Salloum R.G. (2015). The global epidemiology of waterpipe smoking. Tob. Control.

[14] Salloum R.G., Thrasher J.F., Getz K.R., Barnett T.E., Asfar T., Maziak W. (2017). Patterns of Waterpipe Tobacco Smoking Among U.S. Young Adults, 2013–2014. Am. J. Prev. Med..

[15] Akl E.A., Gunukula S.K., Aleem S., Obeid R., Jaoude P.A., Honeine R., Irani J. (2011). The prevalence of waterpipe tobacco smoking among the general and specific populations: A systematic review. BMC Public Health.

[16] Jawad M., Lee J.T., Millett C. (2016). Waterpipe Tobacco Smoking Prevalence and Correlates in 25 Eastern Mediterranean and Eastern European Countries: Cross-Sectional Analysis of the Global Youth Tobacco Survey. Nicot. Tob. Res..

[17] Kheirallah K.A., Alsulaiman J.W., Mohammad H.A., Alzyoud S., Veeranki S.P., Ward K.D. (2016). Waterpipe Tobacco Smoking among Arab Youth; a Cross-Country Study. Ethn. Dis..

[18] Dugas E., Tremblay M., Low N.C.P., Cournoyer D., O’Loughlin J. (2010). Water-Pipe Smoking Among North American Youths. Pediatrics.

[19] Ghafouri N., Hirsch J.D., Heydari G., Morello C.M., Kuo G.M., Singh R.F. (2011). Waterpipe smoking among health sciences university students in Iran: Perceptions, practices and patterns of use. BMC Res. Notes.

[20] Maziak W., Ben Taleb Z., Jawad M., Afifi R., Nakkash R., Akl E.A., Ward K.D., Salloum R.G., Barnett T.E., Primack B.A. (2017). Consensus statement on assessment of waterpipe smoking in epidemiological studies. Tob. Control.

[21] WHO (World Health Organization) (2012). Global Youth Tobacco Survey, Country Fact Sheets: Lebanon (Ages 13–15).

[22] Saade G., Abou Jaoude S., Afifi R., Warren C., Jones N. (2008). Patterns of tobacco use: Results from the 2005 Global Youth Tobacco Survey in Lebanon. East Mediterr. Health J..

[23] Bahelah R., DiFranza J.R., Ward K.D., Eissenberg T., Fouad F.M., Taleb Z.B., Jaber R., Maziak W. (2017). Waterpipe smoking patterns and symptoms of nicotine dependence: The Waterpipe Dependence in Lebanese Youth Study. Addict. Behav..

[24] Lopez A.A., Eissenberg T., Jaafar M., Afifi R. (2017). Now is the time to advocate for interventions designed specifically to prevent and control waterpipe tobacco smoking. Addict. Behav..

[25] Albuquerque M., Starr G., Schooley M., Pechacek T., Henson R. (2003). Advancing Tobacco Control through Evidence-Based Programs.

[26] Lantz P.M., Jacobson P.D., Warner K.E., Wasserman J., Pollack H.A., Berson J., Ahlstrom A. (2000). Investing in youth tobacco control: A review of smoking prevention and control strategies. Tob. Control.

[27] CDC (2014). Best Practices for Comprehensive Tobacco Control Programs—2014.

[28] Vickers K.S., Thomas J.L., Patten C.A., Mrazek D.A. (2002). Prevention of tobacco use in adolescents: Review of current findings and implications for healthcare providers. Curr. Opin. Pediatr..

[29] Jawad M., Jawad S., Waziry R.K., Ballout R.A., Akl E.A. (2016). Interventions for waterpipe tobacco smoking prevention and cessation: A systematic review. Sci. Rep..

[30] Brinker T.J., Stamm-Balderjahn S., Seeger W., Groneberg D.A. (2014). Education Against Tobacco (EAT): A quasi-experimental prospective evaluation of a programme for preventing smoking in secondary schools delivered by medical students: A study protocol. BMJ Open.

[31] Miller M.P., Gillespie J., Billian A., Davel S. (2001). Prevention of smoking behaviors in middle school students: Student nurse interventions. Public Health Nurs..

[32] Reddy K.S., Arora M., Perry C.L., Nair B., Kohli A., Lytle L.A., Stigler M., Prabhakaran D. (2002). Tobacco and alcohol use outcomes of a school-based intervention in New Delhi. Am. J. Health Behav..

[33] Wiehe S.E., Garrison M.M., Christakis D.A., Ebel B.E., Rivara F.P. (2005). A systematic review of school-based smoking prevention trials with long-term follow-up. J. Adolesc. Health.

[34] Thomas R.E., McLellan J., Perera R. (2013). School-based programmes for preventing smoking. Cochrane Database Syst. Rev..

[35] Lovato C.Y., Zeisser C., Campbell H.S., Watts A.W., Halpin P., Thompson M., Eyles J., Adlaf E., Brown K.S. (2010). Adolescent smoking: Effect of school and community characteristics. Am. J. Prev. Med..

[36] Wakefield M.A., Chaloupka F.J., Kaufman N.J., Orleans C.T., Barker D.C., Ruel E.E. (2000). Effect of restrictions on smoking at home, at school, and in public places on teenage smoking: Cross sectional study. BMJ.

[37] Johnson C.A., Cen S., Gallaher P., Palmer P.H., Xiao L., Ritt-Olson A., Unger J.B. (2007). Why smoking prevention programs sometimes fail. Does effectiveness depend on sociocultural context and individual characteristics?. Cancer Epidemiol. Biomark. Prev..

[38] Saraf D.S., Nongkynrih B., Pandav C.S., Gupta S.K., Shah B., Kapoor S.K., Krishnan A. (2012). A systematic review of school-based interventions to prevent risk factors associated with noncommunicable diseases. Asia-Pac. J. Public Health.

[39] Milton M., Maule C., Yee S., Backinger C., Malarcher A., Husten C. (2004). Youth Tobacco Cessation: A Guide for Making Informed Decisions.

[40] Anjum Q., Ahmed F., Ashfaq T. (2008). Knowledge, attitude and perception of water pipe smoking (Shisha) among adolescents aged 14–19 years. J. Pak. Med. Assoc..

[41] Stamm-Balderjahn S., Groneberg D.A., Kusma B., Jagota A., Schönfeld N. (2012). Smoking Prevention in School Students. Deutsch. Aerzteblatt Int..

[42] Quadri M.F., Saleh S.M., Alsanosy R., Abdelwahab S.I., Tobaigy F.M., Maryoud M., Al-Hebshi N. (2014). Effectiveness of an intervention program on knowledge of oral cancer among the youth of Jazan, Saudi Arabia. Asian Pac. J. Cancer Prev..

[43] Essa-Hadad J., Linn S., Rafaeli S. (2015). A Web-Based Program to Increase Knowledge and Reduce Cigarette and Nargila Smoking Among Arab University Students in Israel: Mixed-Methods Study to Test Acceptability. J. Med. Internet Res..

[44] Lipkus I.M., Eissenberg T., Schwartz-Bloom R.D., Prokhorov A.V., Levy J. (2011). Affecting Perceptions of Harm and Addiction among College Waterpipe Tobacco Smokers. Nicot. Tob. Res..

[45] Mays D., Tercyak K.P., Lipkus I.M. (2016). The Effects of Brief Waterpipe Tobacco Use Harm and Addiction Education Messages Among Young Adult Waterpipe Tobacco Users. Nicot. Tob. Res..

[46] Islam F., Salloum R.G., Nakkash R., Maziak W., Thrasher J.F. (2016). Effectiveness of health warnings for waterpipe tobacco smoking among college students. Int. J. Public Health.

[47] Sutfin E.L., Cornacchione Ross J., Lazard A.J., Orlan E., Suerken C.K., Wiseman K.D., Reboussin B.A., Wolfson M., Noar S.M. (2017). Developing a Point-of-Sale Health Communication Campaign for Cigarillos and Waterpipe Tobacco. Health Commun..

[48] CAS (2009). Multiple Indicators Cluster Survey, Round 3 (MICS3): Final Report.

[49] Moher D., Hopewell S., Schulz K.F., Montori V., Gøtzsche P.C., Devereaux P.J., Elbourne D., Egger M., Altman D.G. (2012). CONSORT 2010 explanation and elaboration: Updated guidelines for reporting parallel group randomised trials. Int. J. Surg..

[50] Nakkash R.T., Al Mulla A., Torossian L., Karhily R., Shuayb L., Mahfoud Z.R., Janahi I., Al Ansari A.A., Afifi R.A. (2014). Challenges to obtaining parental permission for child participation in a school-based waterpipe tobacco smoking prevention intervention in Qatar. BMC Med. Ethics.

[51] Glanz K., Rimer B.K., Viswanath K., Glanz K., Rimer B.K., Viswanath K. (2015). Theory, Research, and Practice in Health Behavior and Health Education. Health Behavior: Theory, Research, and Practice.

[52] Bteddini D., Afifi R., Haddad P., Jbara L., Alaouie H., Al Aridi L., Mahfoud Z., Al Mulla A., Nakkash R. (2017). Process evaluation and challenges of implementation of a school-based waterpipe tobacco smoking prevention program for teens in Lebanon. Tob. Prev. Cessat..

[53] Jawad M., Afifi R.A., Mahfoud Z., Bteddini D., Haddad P., Nakkash R. (2016). Validation of a simple tool to assess risk of waterpipe tobacco smoking among sixth and seventh graders in Lebanon. J. Public Health.

[54] Jawad M., Nakkash R.T., Mahfoud Z., Bteddini D., Haddad P., Afifi R.A. (2015). Parental smoking and exposure to environmental tobacco smoke are associated with waterpipe smoking among youth: Results from a national survey in Lebanon. Public Health.

[55] Kandel D.S.E., Kessler R.C. (1976). The Epidemiology of Drug Use among New York State High School Students: Distribution, Trends, and Change in Rates of Use. Am. J. Public Health.

[56] Hansen W.B., Graham J.W. (1991). Preventing alcohol, marijuana, and cigarette use among adolescents: Peer pressure resistance training versus establishing conservative norms. Prev. Med..

[57] Faggiano F., Vigna-Taglianti F., Burkhart G., Bohrn K., Cuomo L., Gregori D., Panella M., Scatigna M., Siliquini R., Varona L. (2010). The effectiveness of a school-based substance abuse prevention program: 18-month follow-up of the EU-Dap cluster randomized controlled trial. Drug Alcohol Depend..

[58] Green L.W., Kreuter M.W. (2005). Health Program Planning: An Educational and Ecological Approach.

[59] McLeroy K.R., Bibeau D., Steckler A., Glanz K. (1988). An ecological perspective on health promotion programs. Health Educ. Q..

[60] Wong S.L., Shields M., Leatherdale S., Malaison E., Hammond D. (2012). Assessment of validity of self-reported smoking status. Health Rep..

[61] WHO (2017). WHO Report on the Global Tobacco Epidemic, 2017: Monitoring Tobacco Use and Prevention Policies.

